# Improved branch and bound algorithm for detecting SNP-SNP interactions in breast cancer

**DOI:** 10.1186/2043-9113-3-4

**Published:** 2013-02-14

**Authors:** Li-Yeh Chuang, Hsueh-Wei Chang, Ming-Cheng Lin, Cheng-Hong Yang

**Affiliations:** 1 Department of Chemical Engineering & Institute of Biotechnology and Chemical Engineering, I-Shou University, No.1, Sec. 1, Syuecheng Rd. Dashu District, Kaohsiung 84001, Taiwan; 2 Department of Biomedical Science and Environmental Biology, Kaohsiung Medical University, Kaohsiung 80708, Taiwan; 3 Graduate Institute of Natural Products, College of Pharmacy, Kaohsiung Medical University, Kaohsiung 80708, Taiwan; 4 Cancer Center, Kaohsiung Medical University Hospital, Kaohsiung Medical University, Kaohsiung 80708, Taiwan; 5 Department of Electronic Engineering, National Kaohsiung University of Applied Sciences, 415 Chien-Kung Road, Kaohsiung 80778, Taiwan

## Abstract

**Background:**

Single nucleotide polymorphisms (SNPs) in genes derived from distinct pathways are associated with a breast cancer risk. Identifying possible SNP-SNP interactions in genome-wide case–control studies is an important task when investigating genetic factors that influence common complex traits; the effects of SNP-SNP interaction need to be characterized. Furthermore, observations of the complex interplay (interactions) between SNPs for high-dimensional combinations are still computationally and methodologically challenging. An improved branch and bound algorithm with feature selection (IBBFS) is introduced to identify SNP combinations with a maximal difference of allele frequencies between the case and control groups in breast cancer, i.e., the high/low risk combinations of SNPs.

**Results:**

A total of 220 real case and 334 real control breast cancer data are used to test IBBFS and identify significant SNP combinations. We used the odds ratio (*OR*) as a quantitative measure to estimate the associated cancer risk of multiple SNP combinations to identify the complex biological relationships underlying the progression of breast cancer, i.e., the most likely SNP combinations. Experimental results show the estimated odds ratio of the best SNP combination with genotypes is significantly smaller than 1 (between 0.165 and 0.657) for specific SNP combinations of the tested SNPs in the low risk groups. In the high risk groups, predicted SNP combinations with genotypes are significantly greater than 1 (between 2.384 and 6.167) for specific SNP combinations of the tested SNPs.

**Conclusions:**

This study proposes an effective high-speed method to analyze SNP-SNP interactions in breast cancer association studies. A number of important SNPs are found to be significant for the high/low risk group. They can thus be considered a potential predictor for breast cancer association.

## Background

At present, identifying SNP-SNP interactions in genome-wide case–control studies is computationally and methodologically challenging [1]. To better understand the complex disease characteristics in case–control studies, we extended previous research of a breast cancer study and simultaneously explored single nucleotide polymorphism (SNP) combinations in low and high risk groups [2]. In complex diseases and cancers, joint genetic effects (epistasis) across the whole genome need to be considered. In a recent study, Phillips identifies three types of epistasis: compositional epistasis, statistical epistasis and functional epistasis [3]. Compositional epistasis blocks the effect of one allele by another at a different locus, statistical epistasis constitutes a statistical deviation from the additive effects of two loci on the phenotype, and functional epistasis addresses molecular interactions [3,4].

Many methods have been developed to detect epistasis on the basis of a statistical definition to explore gene-gene interactions or SNP-SNP interactions (epistasis) in complex diseases; these include logic regression [5,6], Multifactor-Dimensionality Reduction (MDR) [7], Polymorphism Interaction Analysis (PIA) [8], Bayesian model selection [9], SNPruler [10], random jungle [11], genetic algorithms [12] and other methods [13-16]. The challenges posed by traditional parametric statistical methods (e.g., logistic regression models) have been detailed in Hahn [6]. The MDR method is inspired by the combinatorial partitioning method, in which a data-reduction method effectively reduces the genotype predictors from *n* dimensions to one dimension. However, the computational load can be excessive when dealing with more than 10 polymorphisms [17]. PIA uses a case-based exclusion for missing SNP data, i.e., only those subjects for which all SNPs are identified (in a particular combination) are used in the analysis. SNPruler is a statistical method for identifying SNP combinations; it uses the Chi-square test to design the bound in the original Branch and Bound algorithm. Unlike our study, which focuses on the difference between cases and controls, SNPruler focuses on the ratio between cases and controls. Although these methods are widely used, they can still be improved upon. As a test data set increases in size, the run time increases exponentially with the order of interaction. However, few studies address SNP-SNP interactions for multiple SNPs. Hence, when a data set is sufficiently large, selecting an appropriate method becomes important.

This study proposes a method based on statistical epistasis and an improved branch and bound algorithm combined with feature selection (IBBFS) to explore combinations of SNP-SNP interactions in a breast cancer association study. The proposed method can reduce the search time and accurately determine the difference between cases and controls in low and high risk groups. Finally, we use the odds ratio (*OR*) as a quantitative measure to assess combinations of SNPs in the case–control studies. The odds ratio is a commonly-used statistic that expresses the strength of association between exposure and disease [18-20]. Experimental results show that the IBBFS method can determine risk factors in breast cancers.

## Results

### Identification of best SNP-SNP interaction combinations with maximal difference between cases and controls

The IBBFS method was used to find the best combination of SNPs in the high and low risk groups, with the best combinations of two-SNP interaction results shown in Table 1. We sorted the combinations of the two-SNP results and selected the top three maximum difference combinations in the low and high risk groups. In Table 1, the six specific SNP combinations with their corresponding genotypes (i.e., the SNPs (4, 7) with genotype (2–3) [CXCL12 (rs1801157)-AG]-[KITLG (rs10506957)-CC]) showed a maximal difference value of 7 between the 4 samples in the control data and the 11 samples in the high risk case data groups. The SNPs (3, 4) with genotype (1–1), [CXCR4 (rs2228014)-CC]-[CXCL12 (rs1801157)-GG], showed a maximal difference value of 68 between the 137 samples in the control data and the 69 samples in low risk case data groups. SNPs (4, 7) with genotype (2–3) and SNPs (3, 4) with genotype (1–1) are statistically significant because their *p*-value is smaller than 0.05. We then extended the best results of the two-SNP combinations to three SNPs. In this way all combinations are extended until the maximum number of SNPs was reached.

**Table 1:** Estimated best combinations of two SNPs on the occurrence of breast cancer

**Combined SNP number (specific SNPs)**		**SNP Genotypes**	**Control number / Case number**	**CC**	**SN**	**SP**	**Average**	**Odds Ratio (95%CI)**	***p*-value**
High-risk	Two SNPs	Other	330/209						
	SNPs (4, 7)	2-3	4/11	0.616	0.050	0.988	0.551	4.342 (1.259-16.394)	0.013
			(Diff. = 7)						
	Two SNPs	Other	327/209						
	SNPs (4, 6)	3-2	7/11	0.610	0.050	0.979	0.546	2.459 (0.867-7.143)	0.084
			(Diff. = 4)						
	Two SNPs	Other	289/172						
	SNPs (3, 5)	2-1	45/48	0.608	0.218	0.865	0.564	1.792 (1.118-2.875)	0.014
			(Diff. = 3)						
Low-risk	Two SNPs	Other	197/151						
	SNPs (3, 4)	1-1	137/69	0.480	0.314	0.589	0.461	0.657 (0.452-0.955)	0.025
			(Diff. = 68)						
	Two SNPs	Other	226/174						
	SNPs (3, 7)	1-2	108/46	0.491	0.209	0.676	0.459	0.553 (0.364-0.839)	0.004
			(Diff. = 62)						
	Two SNPs	Other	223/168						
	SNPs (1, 3)	2-1	111/52	0.496	0.236	0.668	0.467	0.622 (0.415-0.931)	0.017
			(Diff. = 59)						

^NOTE -^ Other: The reference group, indicating the union of all other possible two to seven SNP combinations; N.E: Not estimable; CC: Correct; SN: Sensitivity; SP: Specificity.

Finally, we used IBBFS to find the best-performing combinations of three or more SNPs, with results shown in Tables 2 and 3. Table 2 shows the maximum difference combinations (two to seven SNPs) for the high risk category. These respective combinations are SNPs (4, 7) with genotypes (2–3) and an *OR* of 4.342, SNPs (3, 5, 6) with genotypes (2-1-1) and an *OR* of 2.384, SNPs (3, 4, 5, 6) with genotypes (2-1-1-1) and an *OR* of 3.024, and SNPs (1, 3, 4, 5, 6) with genotypes (1-2-1-1-1) and an *OR* of 3.133. These two-to-five SNP combinations are statistically significant with a *p*-value smaller than 0.05. However, for combinations of six SNPs and combinations of seven SNPs, the *p*-value is greater than 0.05. In Table 3, the results for SNPs (3, 4) with genotypes (1–1), SNPs (1, 3, 5) with genotypes (2-1-1), SNPs (1, 2, 3, 4) with genotypes (2-2-1-1), and SNPs (1, 2, 3, 4, 5) with genotypes (2-2-1-1-1) all have a *p*-value smaller than 0.05. For all other combinations of SNPs the *p*-value was greater than 0.05. These experimental results prove that the proposed IBBFS method can handle combinations of multiple SNPs and determine the best combination of two to seven SNPs, both the in low and high risk categories.

**Table 2:** Estimated best combinations of SNPs on the occurrence of breast cancer in the high risk group

**Combined SNP number (specific SNPs)**	**SNP Genotypes**	**Control number / Case number**	**CC**	**SN**	**SP**	**Average**	**Odds Ratio (CI)**	***p*-value**
Two SNPs	Other	330/209						
SNPs (4, 7)	2-3	4/11	0.615	0.050	0.988	0.551	4.342 (1.259-16.934)	0.013
		(Diff. = 7)						
Three SNPs	Other	314/191						
SNPs (3, 5, 6)	2-1-1	20/29	0.619	0.132	0.940	0.564	2.384 (1.263-4.518)	0.005
		(Diff. = 9)						
Four SNPs	Other	325/203						
SNPs (3, 4, 5, 6)	2-1-1-1	9/17	0.617	0.077	0.973	0.556	3.024 (1.246-7.491)	0.008
		(Diff. = 8)						
Five SNPs	Other	329/210						
SNPs (1, 3, 4, 5, 6)	1-2-1-1-1	5/10	0.612	0.045	0.985	0.547	3.133 (0.969-10.680)	0.031
		(Diff. = 5)						
Six SNPs	Other	332/214						
SNPs (1, 2, 3, 5, 6, 7)	1-2-2-1-1-2	0/4	0.610	0.018	1.000	N.E		
		(Diff. = 4)						
Seven SNPs	Other	333/216						
SNPs (1, 2, 3, 4, 5, 6, 7)	2-2-2-1-1-1-1	1/4	0.608	0.014	0.997	0.540	6.167 (0.648-145.871)	0.084
		(Diff. = 3)						

^NOTE -^ Other: The reference group, indicating the union of all other possible two to seven SNP combinations; N.E: Not estimable; CC: Correct; SN: Sensitivity; SP: Specificity.

**Table 3:** Estimated best combinations of SNPs on the occurrence of breast cancer in the low risk group

**Combined SNP number (specific SNPs)**	**SNP Genotypes**	**Control number / Case number**	**CC**	**SN**	**SP**	**Average**	**Odds Ratio (CI)**	***p*-value**
Two SNPs	Other	197/151						
SNPs (3, 4)	1-1	137/69	0.480	0.314	0.600	0.465	0.657 (0.452-0.955)	0.025
		(Diff. = 68)						
Three SNPs	Other	260/189						
SNPs (1, 3, 5)	2-1-1	74/31	0.525	0.141	0.778	0.481	0.576 (0.355-0.934)	0.020
		(Diff. = 43)						
Four SNPs	Other	294/207						
SNPs (1, 2, 3, 4)	2-2-1-1	40/13	0.554	0.059	0.880	0.498	0.462 (0.228-0.919)	0.018
		(Diff. = 27)						
Five SNPs	Other	310/215						
SNPs (1, 2, 3, 4, 5)	2-2-1-1-1	24/5	0.569	0.023	0.928	0.507	0.300 (0.099-0.846)	0.011
		(Diff. = 19)						
Six SNPs	Other	323/218						
SNPs (1, 2, 3, 4, 5, 6)	2-2-1-1-1-2	11/2	0.587	0.009	0.967	0.521	0.269 (0.041-1.301)	0.070
		(Diff. = 9)						
Seven SNPs	Other	325/219						
SNPs (1, 2, 3, 4, 5, 6, 7)	2-2-1-1-1-2-1	9/1	0.588	0.005	0.973	0.522	0.165 (0.008-1.277)	0.098
		(Diff. = 8)						

^NOTE -^ Other: The reference group, indicating the union of all other possible two to seven SNP combinations; N.E: Not estimable; CC: Correct; SN: Sensitivity; SP: Specificity.

### Analysis of combinations of SNP (4, 7) and combinations of SNP (3, 4) in breast cancer

**Figure 1:**
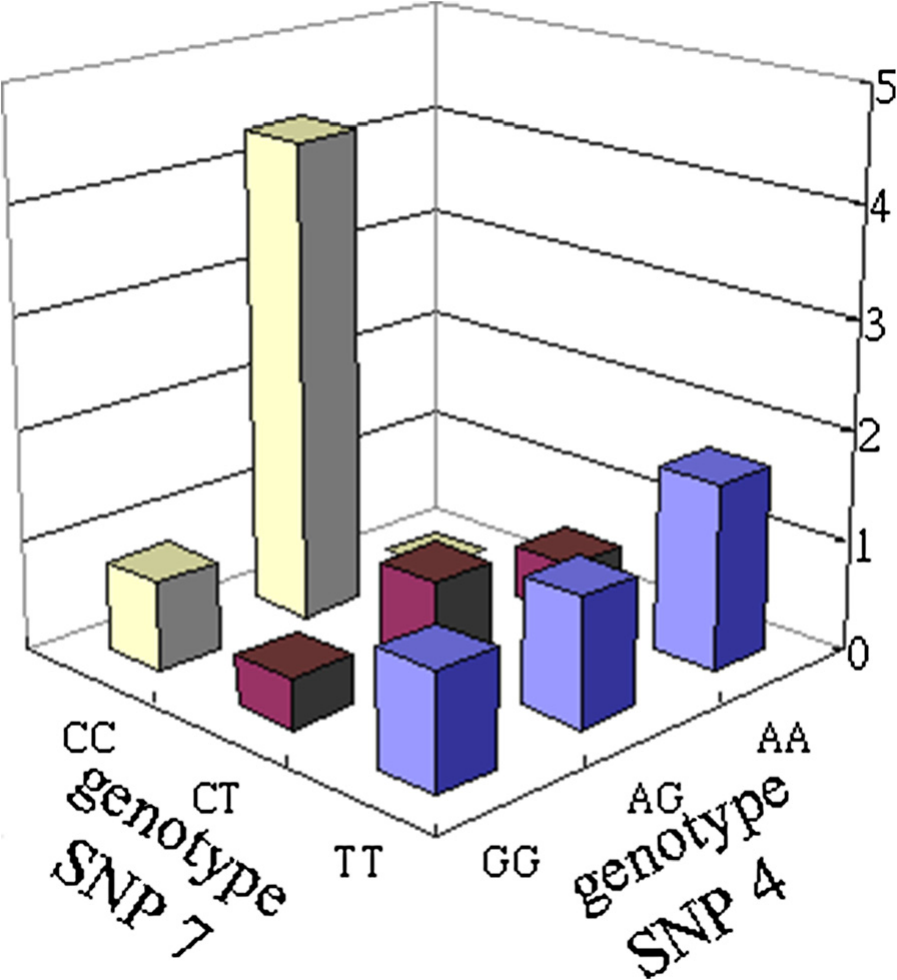
SNPs (4, 7) with their corresponding genotypes CXCL12-rs1801157 and KITLG-rs10506957.

First, we analyzed the high risk combination of SNP (4, 7) in breast cancer. Information related to the SNP (4, 7) combination and the *OR* results are shown in Table 4, while Figure 1 displays a bar graph illustrating the *OR* value. Two SNPs are shown in a 3 × 3 table that represents nine state combinations. IBBFS shows that SNP (4, 7) with genotype (2–3) with a maximal *OR* value of 4.342 (*p*-value < 0.05) has a maximal difference of 7 between the case and control groups. We observe that, for other combinations of SNP (4, 7) with genotype (1–2), the *OR* value is at a minimum and the *p*-values are statistically significant (*p*-value < 0.05). Hence, only two combinations of SNP (4, 7) in the 3 × 3 table are statistically significant (*p*-value < 0.05).

**Table 4:** **Odds ratio (*OR *) (95% CI) (*p *-value) for SNP interactions in SNP (4, 7) combinations**

		**Statistics**	CXCL12^*^		
			**Genotypes (X)**		
			**GG**	**AG**	**AA**
KITLG^*^	TT	*OR*^*a*^	1.047	1.163	1.685
Genotypes (Y)		*CI*	0.709-1.546	0.763-1.773	0.706-4.030
		*p*-value	0.849	0.471	0.214
		*(Ca./Co.)*	(66/97)	(54/73)	(13/12)
	CT	*OR*^*a*^	0.460	0.823	0.499
		*CI*	0.265-0.793	0.503-1.341	0.106-2.034
		*p*-value	0.003	0.484	0.379
		*(Ca./Co.)*	(22/65)	(33/59)	(3/9)
	CC	*OR*^*a*^	0.811	4.342	N.E
		*CI*	0.288-2.221	1.259-16.394	
		*p*-value	0.817	0.013	
		*(Ca./Co.)*	(7/13)	(11/4)	

^NOTE -^ *:SNPs (4, 7) pair with its corresponding genotypes CXCL12-rs1801157- genotype X and KITLG-rs10506957-genotype Y; ^a^: *OR* in reference to the other group (1.000); *CI*: Confidence Interval; *Ca.*: case; *Co.*: control; N.E: Not estimable.

**Figure 2:**
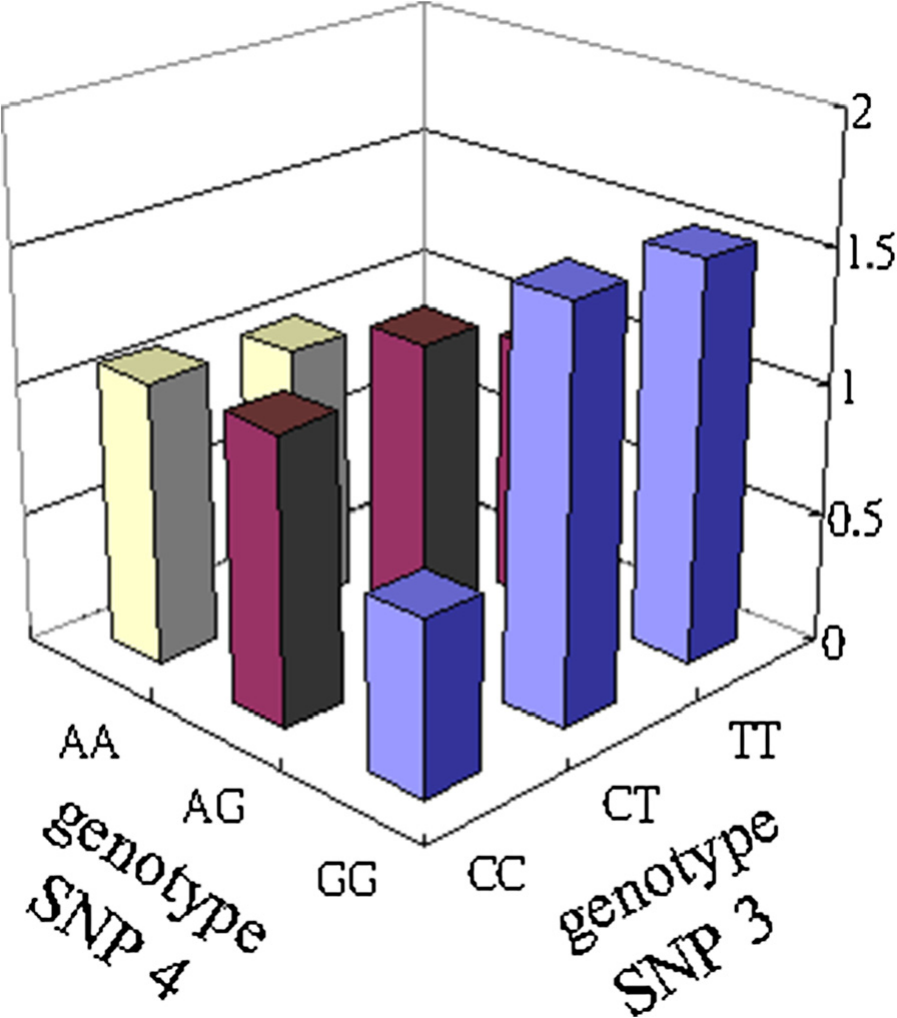
SNPs (3, 4) with their corresponding genotypes CXCR4-rs2228014 and CXCL12-rs1801157.

Secondly, we analyzed the low risk combinations of SNP (3, 4), with the related information and *OR* results shown in Table 5. The bar graphs in Figure 2 illustrate the *OR* value. When the combination of SNP (3, 4) with genotype (1–1) is chosen, the minimum *OR* value is 0.657 (*p*-value < 0.05). Here, the maximum difference also is SNP (3, 4) with genotype (1–1), with a difference of 68 between cases and controls. When considering combinations of more SNPs, the frequently occurring combinations of two SNPs in the following combinations are important.

**Table 5:** **Odds ratio (*OR *) (95% CI) (*p *-value) for SNP interactions in SNP (3, 4) combinations**

		**Statistics**	CXCR4^*^		
			**Genotypes (X)**		
			**CC**	**CT**	**TT**
KITLG ^*^	GG	*OR*^*a*^	0.657	1.557	1.528
Genotypes (Y)		*CI*	0.459-0.940	0.936-2.590	0.414-5.636
		*p*-value	0.025	0.110	0.719
		*(Ca./Co.)*	(69/137)	(33/34)	(4/4)
	AG	*OR*^*a*^	1.092	1.222	1.012
		*CI*	0.757-1.576	0.712-2.098	0.201-5.111
		*p*-value	0.639	0.484	1.000
		*(Ca./Co.)*	(70/100)	(26/33)	(2/3)
	AA	*OR*^*a*^	1.076	1.012	N.E
		*CI*	0.511-2.266	0.303-3.382	
		*p*-value	0.848	1.000	
		*(Ca./Co.)*	(12/17)	(4/6)	

^NOTE -^ *: SNPs (3, 4) pair with its corresponding genotypes CXCR4-rs2228014-genotype X and KITLG-rs10506957-genotype Y; ^a^: *OR* in reference to the other group (1.000); *CI*: Confidence Interval; *Ca.*: case; *Co.*: control; N.E: Not estimable.

### Rank analysis of odds ratios for breast cancer

Tables 2 and 3 show the estimated effects (*CC*, *SN*, *SP*, *OR* and 95% *CI*) of certain specific SNP combinations on the occurrence of breast cancer. These specific SNP combinations (two to seven SNPs) had a 0.657 to 0.165 risk of breast cancer (Table 3). In addition, these specific SNP combinations (two to seven SNPs) also show a higher risk (*OR* > 1) of breast cancer in Table 2. When the *OR* value is larger than 1, the proportion of subjects with breast cancer is higher. On the other hand, when the proportion of subjects with breast cancer is smaller than 1, the *OR* values are lower than 1.

## Discussions

Identification of SNP-SNP interactions (epistasis) is an important task [13,21] when exploring a cancer or disease risk [22-25]. At present, artificial intelligence (AI) algorithms are rarely used to identify SNP-SNP interaction combinations. Although some methods have previously been used to identify SNP combinations (e.g., MDR [7], machine learning [26], particle swarm optimization (PSO) [27], and genetic algorithms (GA) [12], these methods can still be improved upon. MDR, for example, has three distinct disadvantages. First, the method is prone to false positive and false negative errors when the ratio of the number of cases and controls in a combination of genotypes is similar to that in the entire data set. Secondly, MDR binary classification does not provide any quantitative measure of the disease risk for each combination of genotypes, but rather provides a binary measure (high or low) of the disease risk. The MDR method also does not permit comparison of disease risks between different combinations of genotypes [19]. The machine learning method also has a drawback in that it does not provide a quantitative measure of the disease risk for each combination of genotypes. PSO and GA do not guarantee that the best SNP combination can be found (see results in Table 6) since both algorithms use randomly generated initial values and set an arbitrary number of iterations. If the maximum number of iterations set as a termination condition is reached before an optimal solution is found, the PSO and GA algorithms stop the search prematurely. IBBFS, however, can overcome all of the above problems.

**Table 6:** The representative difference of the [Control-Breast Cancer] occurrence value by PSO and GA

**Combination of SNPs**	**SNP Genotype**	**Control (n)/ Breast Case (n)**	**Difference of Control -Breast Case (n)**	**Occurrence**	
				**PSO**	**GA**
SNPs (3,4)	(1–1)	137/69	68	7	7
SNPs (3,7)	(1–2)	108/46	62	2	1
SNPs (3,5)	(1–1)	162/103	59	1	2

^NOTE -^ SNPs (3, 4) represents the combination of SNP_3_-SNP_4_, i.e., rs2228014 - rs1801157. Other combinations follow the same pattern. SNP Genotype (1–1) represent the SNP genotype (CC – GG) combinations. PSO: particle swarm optimization; GA: genetic algorithm.

We focus on understanding the breast cancer risk of functionally-relevant joint effects of combinatorial SNPs within and between different cancer pathways. We calculated the same data set by exhaustive search (ES) using two to seven SNP combinations to find SNP interactions which determine an optimal solution. These calculations were rather time-consuming and the ES method of calculating combinations of SNPs is thus impracticable for large data sets. From a practical standpoint, the main difference between the aforementioned methods is the computational time required to reach an improvement. The IBBFS method found optimal solutions faster for a high order of interaction combinations by cutting off unnecessary paths. IBBFS guarantees that each result contains an optimal solution through the use the integrated feature selection method. The selected number of features is *r = n-m + 1*, where *r* is the number of features used, and *n* and *m* are the total number of SNPs and the number of selected SNPs, respectively. Examples of the ES and IBBFS calculations are respectively shown in Additional file 1: Figure S1 and Additional file 1: Figure S2. The number of possible solutions calculated by ES was 30229, whereas IBBFS reduced this number to 348. IBBFS is thus better suited to deal with large data sets. IBBFS allows for the investigation of an almost unlimited number of SNP combinations, whereas traditional algorithms are rather limited. The experiments show that IBBFS has great potential for the identification of complex biological relationships among cancer processes during the development of breast cancer.

## Conclusion

This study focused on the selection of SNP combinations that give a maximal difference between case and control groups. Evaluating a large number of SNPs associated with a disease requires a strategy for focusing on only a select number of complex interactions. IBBFS was used on complex SNP-SNP interactions and was demonstrated to provide the best SNP-SNP interactions for predicting breast cancer susceptibility. The odds ratio (*OR*) was used as a quantitative measure of the breast cancer risk. Experimental results indicate that the proposed IBBFS method can identify the complex interactions of the tested SNPs both in the low and high risk groups. In the future, the IBBFS method can potentially be applied to SNP-SNP interactions (epistasis) in other association studies.

## Methods

### Data sets

The data set was provided by Lin et al. and includes breast cancer data, SNPs, personal information and clinical data [2]. This study continues research from a previous study with 220 case and 334 control breast cancer data sets. The case control study was conducted at the Kaohsiung Medical University in Taiwan. The data sets were collected from female patients who came to said hospital for routine physical checkups or distinctive minor operations. The SNP name, the number of cases, the number of controls, and related information is shown in Table 7.

**Table 7:** Baseline characteristics of breast cancer cases and controls

**SNP (Genes)**	**Chr.**	**SNP Genotype**	**Control no. /Case no.**	**Scoring function**					***p*-value**
				**CC**	**SN**	**SP**	**Average**	**Odds Ratio**	
1. rs12812942	12	1-AA	174/128						
(CD4)		2-AT	141/76	0.482	0.372	0.552	0.469	0.733 (0.503-1.068)	0.10
		3-TT	19/16	0.564	0.111	0.902	0.526	1.145 (0.536-2.438)	0.72
2. rs3136685	17	1-GG	107/77						
(CCR7)		2-AG	180/114	0.462	0.587	0.373	0.474	0.880 (0.594-1.304)	0.57
		3-AA	47/29	0.523	0.274	0.695	0.357	0.857 (0.478-1.536)	0.68
3. rs2228014	2	1-CC	254/151						
(CXCR4)		2-CT	73/63	0.586	0.294	0.777	0.552	1.452 (0.962-2.191)	0.07
		3-TT	7/6	0.622	0.382	0.973	0.659	1.442 (0.421-4.880)	0.57
4. rs1801157	10	1-GG	175/106						
(CXCL12)		2-AG	136/98	0.530	0.480	0.562	0.524	1.189 (0.822-1.723)	0.37
		3-AA	23/16	0.597	0.131	0.884	0.537	1.149 (0.550-2.387)	0.73
5. rs3025039	6	1-CC	211/155						
(VEGF)		2-CT	117/59	0.498	0.276	0.643	0.472	0.687 (0.463-1.016)	0.05
		3-TT	6/6	0.574	0.037	0.972	0.528	1.361 (0.381-4.870)	0.77
6. rs2287074	16	1-GG	164/113						
(MMP2)		2-AG	139/93	0.505	0.451	0.541	0.499	0.971 (0.670-1.408)	0.93
		3-AA	31/14	0.553	0.110	0.841	0.510	0.655 (0.316-1.347)	0.25
7. rs10506957	12	1-TT	182/133						
(KITLG)		2-CT	133/69	0.486	0.342	0.578	0.469	0.709 (0.484-1.042)	0.08
		3-CC	19/18	0.568	0.119	0.905	0.531	1.296 (0.622-2.700)	0.08

^NOTE -^ Chr: Chromosome; Control no: number of control; Case no: number of case; CC: Correct; SN: Sensitivity; SP: Specificity.

### Branch and bound algorithm

The branch and bound algorithm (BB) is a divide-and-conquer approach used to solve global optimization issues [28]. The concept of a BB is based on constructing a search tree. Only feasible solutions are used and explicitly evaluated to detect optimal solutions. A BB algorithm requires two steps. First, a branching procedure is used to define the tree structure (the search tree). Then a bounding procedure that computes upper and lower bounds for the evaluation value (evaluation nodes) is implemented. If the next node (lower bound) in the series does not conform to the evaluation value (set bound value), the node is cut off. Compared to exhaustive search (ES), traditional BB algorithms do not guarantee that enough subtrees are cut off to keep the total number of criteria computations lower than in the ES method [29]. Under most circumstances, a traditional BB algorithm is faster than an exhaustive search. However, many redundant searches are still conducted in a BB algorithm [30]. To overcome this problem, Somol proposed the fast branch and bound algorithm [28] and Chen proposed an improved branch and bound algorithm for optimal feature subset selection [30]. Branch and bound algorithms have been successfully applied in many fields, such as predicting drug-like compounds [31], analysis of protein–protein interactions [32], feature selection problems [29] and data mining problems [33,34]. In addition, branch and bound performance may be weaker under the following conditions: (1) Nearer to the root, the criterion value computation is usually slower (evaluated feature subsets are larger) and (2) nearer to the root, subtree cut-offs are less frequent (higher criterion values of larger subsets are compared to the bound, which is updated in the leaves) [28]. A possible solution tree is introduced in Additional file 1: Figure S3 and a BB algorithm flowchart is shown in Additional file 1: Figure S4.

### Improved branch and bound algorithm with feature selection

As previously stated, traditional BB algorithms that search for all possible combinations are impractical since the number of combinations increases exponentially as the dimensionality increases [30]. Hence, we propose the use of a BB algorithm combined with a feature selection technique to reduce the necessary calculation time. Feature selection algorithms are special from a theoretical perspective. It can be shown that optimal feature selection for supervised learning problems requires an exhaustive search of all possible subsets of features of the chosen cardinality [30]. A large number of features is thus impractical. By using a subset of features, the processing time required by the classification process can be reduced. This improved branch and bound algorithm has several advantages when combined with feature selection (IBBFS). It not only reduces the search time but, more importantly, also sorts the results into low and high risk groups (discussed in the bound evaluation section). The IBBFS algorithm is very efficient because it avoids exhaustive searches (ES) by rejecting suboptimal subsets. It also guarantees that a selected subset yields the best global value. A flowchart of this process is shown in Additional file 1: Figure S5. The IBBFS pseudo-code is given in below.

### IBBFS pseudo-code

B: Defined as 0.; R: Number of features used.; N: Total number of SNPs.; M: Total number of selected SNPs.; AVAIL: List of available feature values that LIST(*m*) can assume.; LIST(m): List of the features that can be assumed at level *m*.; Φ: Empty set.

Step 1: Initialize

Level *m*=1, AVAIL ={node_*m*_ 1, node_*m*_ 2, node_*m*_*j*, _…_, node_*m*_*r* | node_*m-1*_ ≠Φ,

*r*=(*n*-*m*+1) × (*n*-(*m*-1)), *j* is the *j*th node}

Step 2: Generate branch

LIST(*m*)={AVAIL | select top *r* node based on their bound value}

If LIST(*m*) =Φ, go to step 5.

Step 3: Select node

Select the rightmost node in LIST(*m*), i.e., if node_*m*_*j*=max(LIST(*m*)) remove the

rightmost node in LIST(*m*)

Step 4: Calculate bound value

If bound(node_*m*_*j*) >*B*, return node_*m*_*j* to AVAIL and go to Step 5.

If last node in level *m*

If level *m*= higher level, go to Step 6, otherwise, *m*=*m*+1 and go to Step 2.

Step 5: Backtrack

If LIST(*m*) is empty, set *m*=*m*-1. If *m*=0, terminate the algorithm,

otherwise, go to Step 3.

Step 6: Higher level,

Sort nodes_*m*_ based on bound value

Return best node_*m*_.

IBBFS uses top-down and right-left search strategies together with backtracking. We define the update bound value as 0, which means that, if the number of cases and controls is 0, the node should be not explored. If the bound value at a node *j* at level *m* is larger than the current bound value *B*, then the paths originating from that node to the bottom of the tree should still be explored. We select the top *r* node based on the bound value for exploration to the next level. Omitting the evaluation of bound values for a set of successor nodes (i.e., *j* < *r* at some parent nodes) is key to an efficient IBBFS. Backtracking is used until all successors or nodes and paths with bounds larger than the current bound value B have been searched. The computational savings in the IBBFS occur when the bound value at a node *j* at a higher level in the tree is the best value.

### Bound evaluation

Statistical epistasis is a population phenomenon that depends on allele frequencies present in a specific population [35]. This study uses the maximum difference of allele frequencies between case and control groups to evaluate the bound value. A large difference in the bound value indicates that certain SNP and genotype combinations are more likely to occur in breast cancer, whereas other combinations are associated with a low cancer risk. We divided the bound calculation into two separate steps: 1) The total number of SNP combinations in the control data set is calculated and 2) the total number of SNP combinations in the case data set is calculated. Subsequently, Eq. (1) is used to determine the bound value of each combination to find the maximum difference.

(1) $$\mathrm{bound}\left(i\right)=\sum_{n=1}^{N}\left(\mathrm{Check}\_\mathrm{contro}l_{n}\left(i\right)\right)-\sum_{c=1}^{C}\left(\mathrm{Check}\_\mathrm{cas}e_{c}\left(i\right)\right)$$

In Eq. (1), *N* represents the number of samples in the control data, and *C* represents the number of samples in the case data. *Check_control*_*n*_ (*i*) and *Check_case*_*c*_ (*i*) are respectively checked as to whether or not the node *i* (i.e., SNP combination) matches the *n* sample in the control data and the *c* sample in the case data. If a match occurs, *Check_control*_*n*_ (*i*)/ *Check_case*_*c*_ (*i*) is set to 1, otherwise, it is set to 0. $\sum_{n=1}^{N}\left(\mathrm{Check}\_\mathrm{contro}l_{n}\right)$ represents the sum of the *Check_control*_*n*_ (*i*) from 1 to *N*, and $\sum_{c=1}^{C}\left(\mathrm{Check}\_\mathrm{cas}e_{c}\right)$ represents the sum of the *Check_case*_*c*_ (*i*) from 1 to *C*. If the positive maximum bound value is selected as a feature in the next combination, then the respective *OR* value indicates a low cancer risk. On the other hand, if the negative maximum bound is selected as a feature in the next combination, then the respective *OR* value is associated with a high cancer risk. The supplementary example illustrates how the bound values are calculated.

For example, assume that *SNPs (3, 4)* with genotype (1–1) are the best SNP combination. SNP_3_ (rs2228014) has the three genotypes CC, CT, and TT, which can be respectively represented as 1, 2, and 3, and SNP_4_ (rs1801157) has the three genotypes GG, AG, and AA, which can also be respectively represented as 1, 2, and 3. We compute the number that matches the condition of the SNPs and genotypes for the case and control data. First, we calculate the control number for *SNP*_*3*_ with genotype 1 and *SNP*_*4*_ with genotype 1. The number of controls that independently match *SNP*_*3*_ with genotype 1 and *SNP*_*4*_ with genotype 1 are 254 and 175, respectively. The number of controls that match SNP (3, 4) with genotype (1–1) is thus 137. Secondly, we calculate the number of cases independently matching *SNP*_*3*_ with genotype 1 and *SNP*_*4*_ with genotype 1 as 151 and 106, respectively. The number of cases that match SNP (3, 4) with genotype (1–1) is thus 69. According to Eq. (1), the bound value is determined by subtracting 69 from 137, thus giving 68.

### Performance measurement

We used four common criteria to determine the best combinations of SNPs related to the cancer risk, namely the correctness (*CC*), the sensitivity (*SN*), the specificity (*SP*) and the odds ratio (*OR*) [8]. The odds ratio has become widely used in epidemiology and case control studies. It is a commonly-used statistic that expresses the strength of association between an exposure and a disease [36,37] due to the following three facts: 1) *OR* provides an estimate (i.e., a confidence interval) for the relationship between two binary variables; 2) it allows us to examine the effects of other variables on that relationship via logistic regression; and 3) *OR* is very convenient for interpretation of case–control studies [18]. It corresponds to the effect of each specific SNP–SNP interaction combination on the occurrence of breast cancer. The four criteria are defined in Eqs. (2–5), and the calculation processes are shown in Additional file 1: Figure S6. Statistical analysis was carried out using http://statpages.org/ctab2x2.html.

(2) $$\mathrm{CC}=\frac{\mathrm{TP}+\mathrm{TN}}{\mathrm{TP}+\mathrm{FN}+\mathrm{FP}+\mathrm{TN}}$$

(3) $$\mathrm{SN}=\frac{\mathrm{TP}}{\mathrm{TP}+\mathrm{FN}}$$

(4) $$\mathrm{SP}=\frac{\mathrm{TN}}{\mathrm{TN}+\mathrm{FP}}$$

(5) $$\mathrm{OR}=\frac{\mathrm{TP}\times \mathrm{TN}}{\mathrm{FN}\times \mathrm{FP}}$$

*TP* represents the number of true positives, *TN* represents the number of true negatives, *FN* represents the number of false negatives, and *FP* represents the number of false positives.

### Illustrative example

The proposed IBBFS algorithm with incorporated feature selection selects the most promising solution and then evaluates only the features of the next SNP combinations of this branch. Furthermore, the algorithm is based on the expansion of two-SNP combinations, which means that the two-SNP combination results are used and expanded until the maximum combination (number of SNPs) is reached. For example, if the SNP (1, 2) with genotype (2–2) combinations constitutes the best result (feature), then combinations of three SNPs that contain SNP (1, 2) with genotype (2–2) are found in the next step. The expanded results are SNP (1, 2, 3) with genotype (2-2-1), SNP (1, 2, 3) with genotype (2-2-2), and SNP (1, 2, 3) with genotype (2-2-3). A detailed example is shown in Additional file 1: Figure S7. These expanded results reduce the search time by cutting off unnecessary paths. The update bound value in this study was set to 0, which means that, if the numbers of cases and controls are 0, the node is cut off. In contrast to the BB algorithm, IBBFS only uses selected features (after sorting the results), which allows it to find an optimal solution by cutting off unnecessary pathways. Although the IBBFS algorithm is of a high time complexity for combinations of two SNPs, it performs better for interaction combinations of a high order. After the best SNP combinations are found, the *OR* is used in the next step to evaluate each best SNP combination with regard to the susceptibility risk. A simple IBBFS calculation process is shown in the Additional file 1 section.

In Additional file 1: Figure S3, the different paths from the top to level 1 indicate that level 1 has 4 SNP paths. If the node results are 0 (meaning the number of cases and controls are 0), the node is cut off. Additional file 1: Figure S3 indicates that the y node is cut off at level 1 (two combinations) because it is terminal. Under the same criteria, each new terminal combination is cut off. If the traditional BB algorithm nodes are not cut off, the calculated time complexity equates that of the ES. The ES function is as follows:

(6) $$\sum_{m=2}^{n}C\left(n,m\right)*3^{m}$$

where *n* is the total number of SNPs, and *m* is the number of selected SNP combinations.

When two SNPs are selected and each genotype has three possible state combinations, ES calculates the number of possible solutions as *C*(*4,2*)*3^*2*^*=54*. Based on the aforementioned calculation process, the use of traditional BB algorithms or ES to explore combinations of three, four or more SNPs is impractical since the increased number of combinations exponentially increases the time complexity Simple ES, BB and IBBFS calculation processes are shown in the Additional file 1 section.

## Competing interests

The authors declare that they have no competing financial interests.

## Authors’ contribution

C-HY coordinated and oversaw this study, and modified the manuscript were appropriate. H-WC and L-YC provided the biochemistry background and introduced the bioinformatics needed. M-CL participated in the design of the algorithm, and wrote the program and the manuscript. All authors read and approve the final manuscript.

## Supplementary Material

Additional file 1: Figure S1 Exhaustive search algorithm calculation process. **Figure S2.** Calculation process of the improved branch and bound feature selection (IBBFS) algorithms. **Figure S3.** Branch and bound search tree. **Figure S4.** Flowchart of a branch and bound algorithm. **Figure S5.** Flowchart of the improved branch and bound algorithm (IBBFS). **Figure S6.** Performance calculations. **Figure S7.** Extended SNP combinations. Supplementary example, include a example for calculation of the SNP-SNP interaction, **Figure S8.** Example of a search tree, **Figure S9.** Search tree of two-SNP combinations, **Table S1.** Example data set, **Table S2.** Results for two-SNP combinations, **Table S3.** Results for three-SNP combinations, **Table S4.** Results for four-SNP combinations, **Table S5.** Table of cases and controls, **Table S6.** Common criteria, **Table S7.** Performance calculation. Click here for file
