# Supplementary material for: Improved branch and bound algorithm for detecting SNP-SNP interactions in breast cancer
Source: J Clin Bioinforma. 2013 Feb 14;3:4. doi: 10.1186/2043-9113-3-4 (PMC3626712; doi:10.1186/2043-9113-3-4)
Supplement: Additional file 1: Figure S1 — Exhaustive search algorithm calculation process. Figure S2. Calculation process of the improved branch and bound feature selection (IBBFS) algorithms. Figure S3. Branch and bound search tree. Figure S4. Flowchart of a branch and bound algorithm. Figure S5. Flowchart of the improved branch and bound algorithm (IBBFS). Figure S6. Performance calculations. Figure S7. Extended SNP combinations. Supplementary example, include a example for calculation of the SNP-SNP interaction, Figure S8. Example of a search tree, Figure S9. Search tree of two-SNP combinations, Table S1. Example data set, Table S2. Results for two-SNP combinations, Table S3. Results for three-SNP combinations, Table S4. Results for four-SNP combinations, Table S5. Table of cases and controls, Table S6. Common criteria, Table S7. Performance calculation. [file 2043-9113-3-4-S1.doc]

**SUPPLEMENTARY MATERIAL**

**Figure S1. Exhaustive search algorithm calculation process**


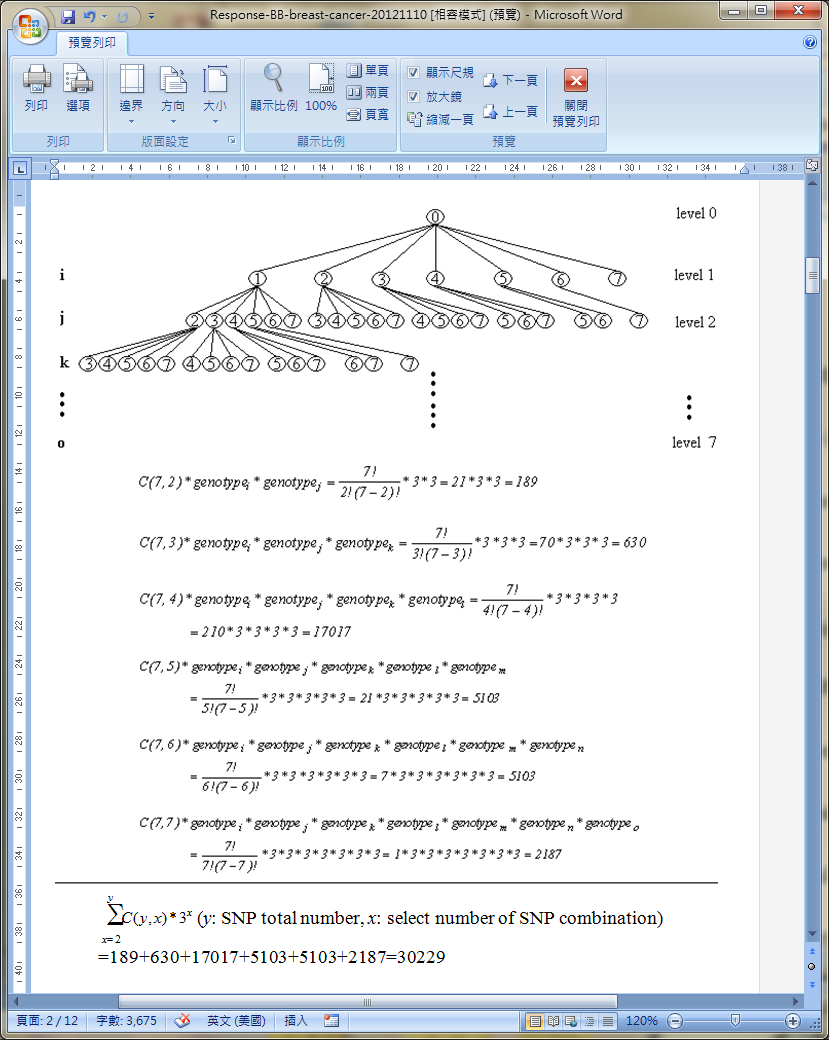


Legend: The symbols i, j, k, l, m, n, and o represent levels 1, 2, 3, 4, 5, 6 and 7 in the branch and bound tree, respectively.

**Figure S2. Calculation process of the improved branch and bound feature selection (IBBFS) algorithms**


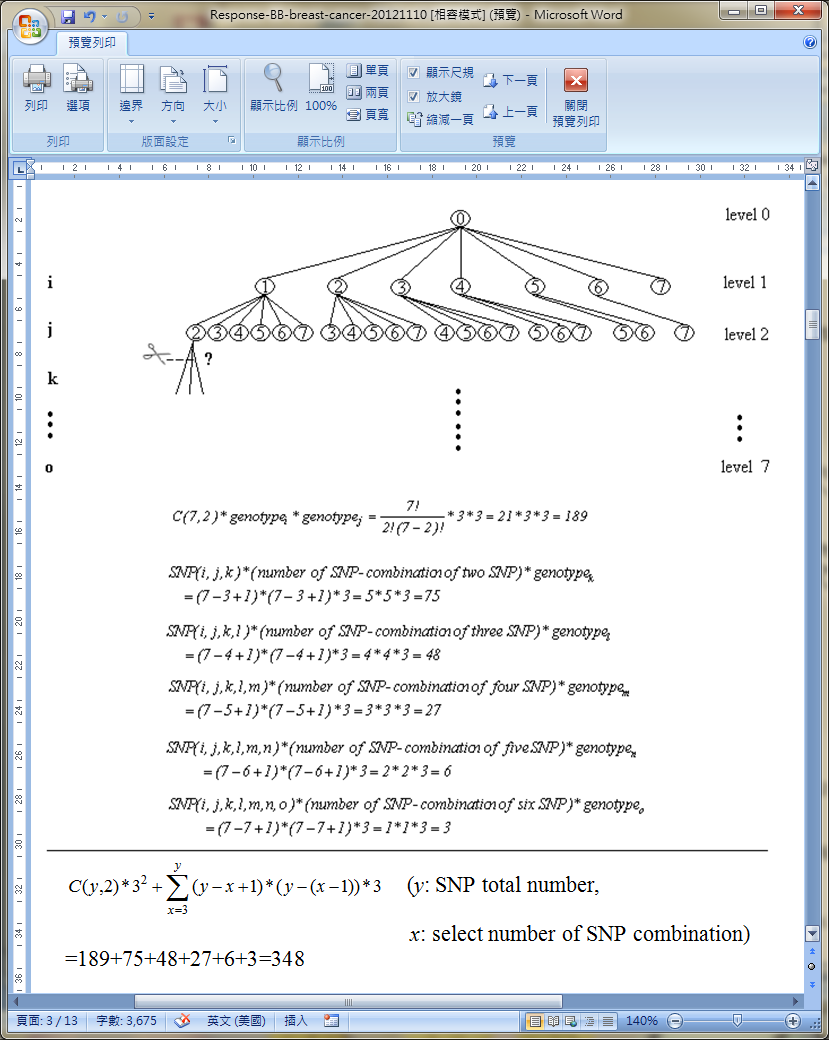


Legend: The symbols i, j, k, l, m, n, and o represent levels 1, 2, 3, 4, 5, 6 and 7 in the branch and bound tree, respectively.

**Figure S3. Branch and bound search tree**


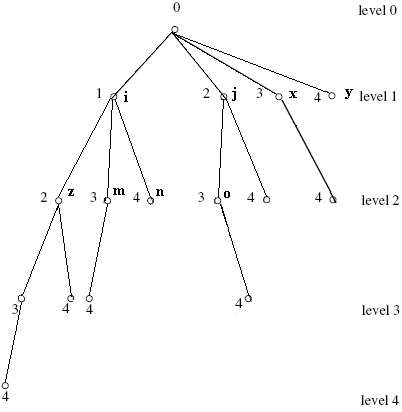


**Figure S4. Flowchart of a branch and bound algorithm**

**Figure S5. Flowchart of the improved branch and bound algorithm (IBBFS)**

**Figure S6. Performance calculations.**

The combination of SNP(3,4) with genotype 1-1 is shown as an example.

**
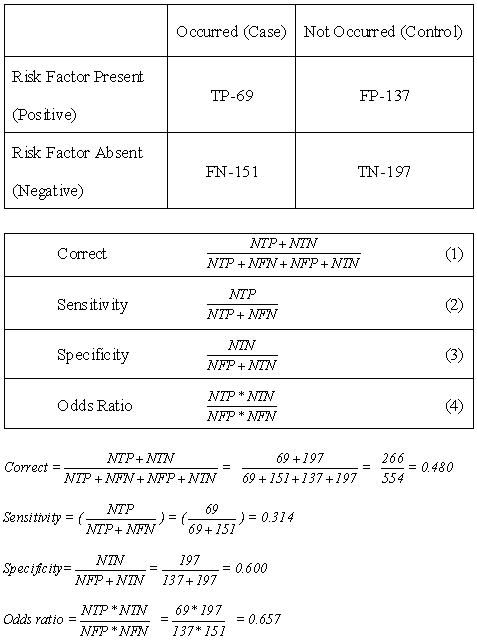
**

**Figure S7. Extended SNP combinations**

If the dataset includes five SNPs, SNP (3, 5) with genotype 2-1 combinations yielded the best result. Combinations of three SNPs that contain the SNP (3, 5) with genotype 2-1 are shown in the next step. The expanded result is shown below:

| **Combined SNP number**  **(specific SNPs)** | **SNP Genotypes** | **Number** | **Combined of Three SNPs** | **SNP Genotypes** |
| --- | --- | --- | --- | --- |
| **Two SNPs**  **SNPs (3, 5)** | **2-1** | 1 | SNPs (1, 3, 5) | 1-2-1 |
|  |  | 2 | SNPs (1, 3, 5) | 2-2-1 |
|  |  | 3 | SNPs (1, 3, 5) | 3-2-1 |
|  |  | 4 | SNPs (2, 3, 5) | 1-2-1 |
|  |  | 5 | SNPs (2, 3, 5) | 2-2-1 |
|  |  | 6 | SNPs (2, 3, 5) | 3-2-1 |
|  |  | 7 | SNPs (3, 4, 5) | 1-2-1 |
|  |  | 8 | SNPs (3, 4, 5) | 2-2-1 |
|  |  | 9 | SNPs (3, 4, 5) | 3-2-1 |

If the sorted results show the SNP (1, 3, 5) with genotype 1-2-1 combinations are best result. In the next step, this study finds the combination of four SNP that will contain the SNP (1, 3, 5) with genotype 1-2-1. The expanded result is shown below:

| **Combined SNP number**  **(specific SNPs)** | **SNP Genotypes** | **Number** | **Combined of Three SNPs** | **SNP Genotypes** |
| --- | --- | --- | --- | --- |
| **Three SNPs**  **SNPs (1, 3, 5)** | **1-2-1** | 1 | SNPs (1, 2, 3, 5) | 1-1-2-1 |
|  |  | 2 | SNPs (1, 2, 3, 5) | 1-2-2-1 |
|  |  | 3 | SNPs (1, 2, 3, 5) | 1-3-2-1 |
|  |  | 4 | SNPs (1, 3, 4, 5) | 1-2-1-1 |
|  |  | 5 | SNPs (1, 3, 4, 5) | 1-2-2-1 |
|  |  | 6 | SNPs (1, 3, 4, 5) | 1-2-3-1 |

If the sorted results show the SNP (1, 3, 4, 5) with genotype 1-2-1-1 combinations are best result. Next step, this study finds the combination of five SNP that will contain the SNP (1, 3, 4, 5) with genotype 1-2-1-1. The expanded result is shown below:

| **Combined SNP number**  **(specific SNPs)** | **SNP Genotypes** | **Number** | **Combined of Three SNPs** | **SNP Genotypes** |
| --- | --- | --- | --- | --- |
| **Four SNPs SNPs (1, 3, 4, 5)** | **1-2-1-1** | 1 | SNPs (1, 2, 3, 4, 5) | 1-1-2-1-1 |
|  |  | 2 | SNPs (1, 2, 3, 4, 5) | 1-2-2-1-1 |
|  |  | 3 | SNPs (1, 2, 3, 4, 5) | 1-3-2-1-1 |

**Illustrative example**

Given are a number of cases and controls of 28 and 30, respectively. The dataset is show in Table S1. The fitness values were calculated with Eq. (1). The search tree is show in Figure S8.

$F_{i}=\sum_{i=1}^{N} (All\_control)-\sum_{i=1}^{N} (All\_case)$ (1)

In Figure S8 we explore the level 1 to level 2 combinations of two SNPs; the results are show in Figure S9 and Table S2. Figure S9 shows the calculated number of solutions is 54, whereas the branch and bound (BB) algorithm only calculated 37 solutions. If these results are used to development combinations of three SNPs, the BB method reduced to 17 the number of combinations in the search. The update bound value in this study was set to 0, which means that if the number of cases and controls is 0, the node is cut off. The exhaustive search (ES) calculation process follows the Eq. (2).

$\sum_{m=2}^{n} C(n,m)*3^{m}$ (2)

With Eq. (2), the ES calculation process yields the following number of solutions:

$$C(4,2)*3^{2}=\frac{4!}{2!(4-2)!}*3^{2}=6*9=54$$

A traditional BB algorithm search for all possible SNP combinations is impractical because the number of combinations increases exponentially as the dimensionality increases. Hence, we combined a BB algorithm with a feature selection technique (IBBFS) to reduce the calculation time and find optimal SNP combinations. The selected number of features is *r=n-m+1*, where *r* is the number of features used, and *n* and *m* are the total number of SNPs and selected SNPs, respectively. Eq. (3) is used for calculations.

*r*=(*n*-*m*+1)*(*n*-(*m*-1)) (3)

Hence, the result for the selected number of features is *r=*(4*-*3*+*1)***(4-(3-1))*=*4. In addition, when the low and high risks are simultaneously considered, the result for the selected numbers of features is 8. As a results ES, BB and IBBFS obtained a number of solutions of 54, 37 and 8, respectively. This indicates that IBBFS significantly reduces the computational time compared to ES and BB. The above calculation processes indicate that the best combinations of SNPs as determined by IBBFS are SNP(2,3) with genotype 2-1, SNP(1,2,3) with genotype 2-2-1 and SNP(1,2,3,4) with genotype 2-2-1-3 in the low risk group, and SNP(2,3) with genotype 1-1, SNP(1,2,3) with genotype 2-1-1 and SNP(1,2,3,4) with genotype 2-1-1-3 in the high risk group (the result are show in Table S2, Table S3 and Table S4). In the next step, the odds ratio (OR) is used as a quantitative measure to estimate the risk. The calculation processes are shown in below.

We calculate the SNP(2,3) with genotype 2-1. Eight cases and 17 with a risk factor are present, and 20 cases and 13 controls with an absent risk factor (Table S5). We use the four common criteria to determine the prediction score (Table S6). Table S7 shows the results of the calculation process. True positives (TP). True negatives (TN). False negatives (FN). False positives (FP).


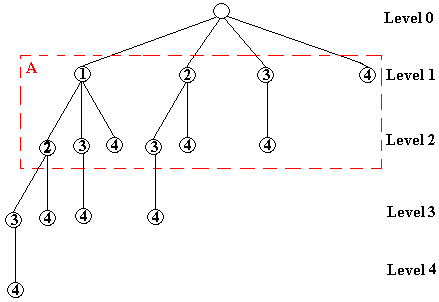


**Figure S8. Example of a search tree**


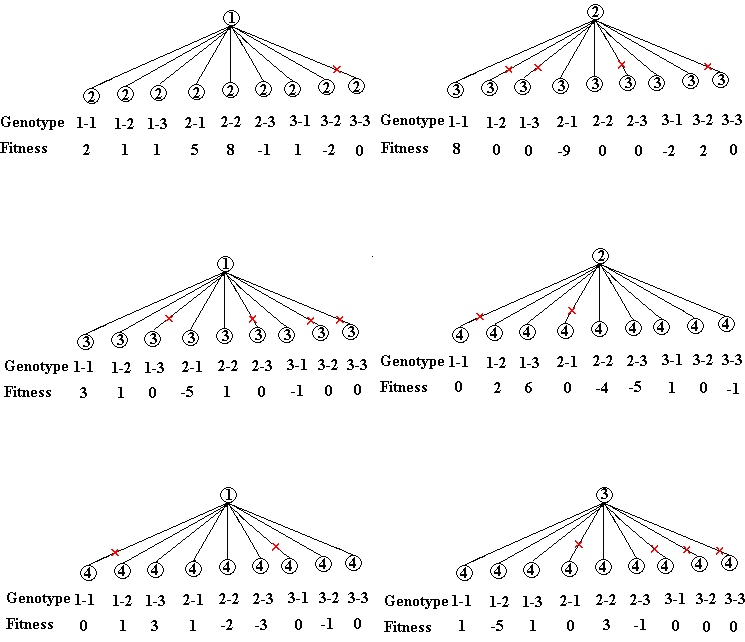


**Figure S9. Search tree of two-SNP combinations**

**Table S1. Example dataset**

| Number of control | | | | | | | | Number of case | | | | | | | |
| --- | --- | --- | --- | --- | --- | --- | --- | --- | --- | --- | --- | --- | --- | --- | --- |
| SNP1 | | SNP2 | | SNP3 | | SNP4 | | SNP1 | | SNP2 | | SNP3 | | SNP4 | |
| G. | N. | G. | N. | G. | N. | G. | N. | G. | N. | G. | N. | G. | N. | G. | N. |
| CC | 0 | AA | 4 | CC | 28 | TT | 0 | CC | 4 | AA | 11 | CC | 24 | TT | 1 |
| CT | 27 | AG | 18 | CT | 2 | TG | 12 | CT | 22 | AG | 9 | CT | 4 | TG | 10 |
| TT | 3 | GG | 8 | TT | 0 | GG | 18 | TT | 2 | GG | 8 | TT | 0 | GG | 17 |
|  | 30 |  | 30 |  | 30 |  | 30 |  | 28 |  | 28 |  | 28 |  | 28 |
| 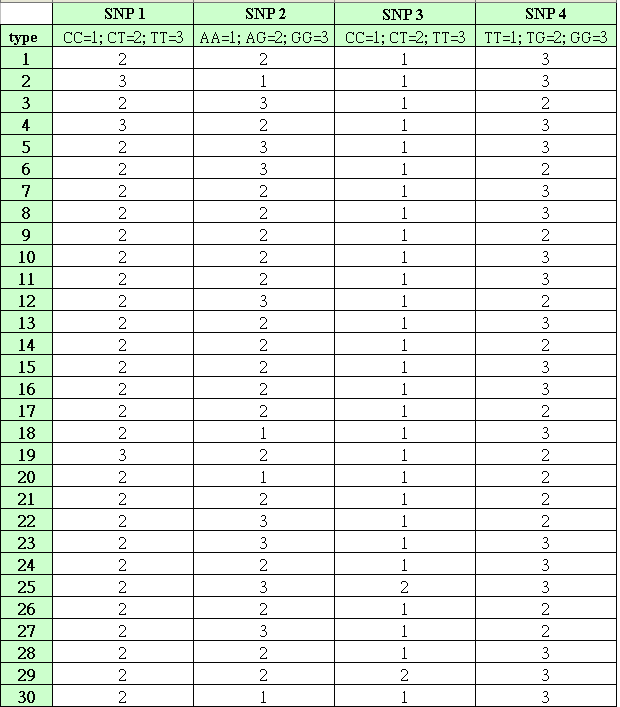 | | | | | | | | 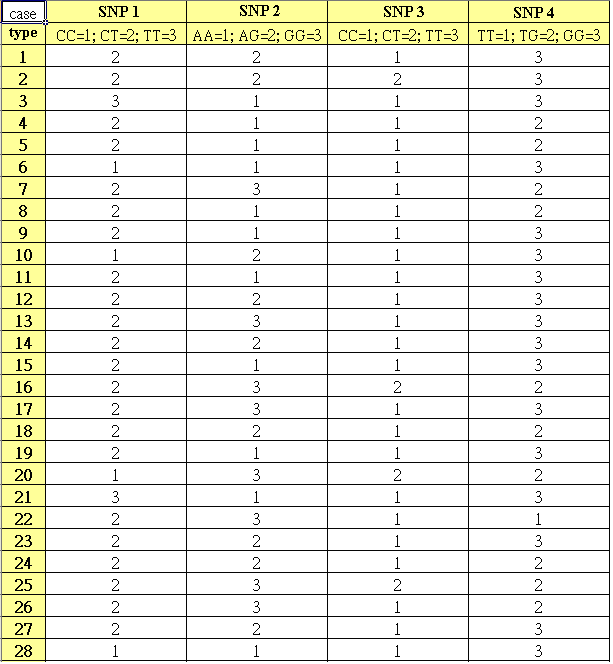 | | | | | | | |

| **Combination of two SNPs** | **SNP**  **Genotype** | **Ca.** | **Co.** | **Diff.** | **Combination of two SNPs** | **SNP**  **Genotype** | **Ca.** | **Co.** | **Diff.** |
| --- | --- | --- | --- | --- | --- | --- | --- | --- | --- |
| SNPs (1,2) | 1-1 | 2 | 0 | 2 | **SNPs (2,3)** | **1-1** | **11** | **3** | **8** |
| SNPs (1,2) | 1-2 | 1 | 0 | 1 | SNPs (2,3) | 1-2 | 0 | 0 | 0 |
| SNPs (1,2) | 1-3 | 1 | 0 | 1 | SNPs (2,3) | 1-3 | 0 | 0 | 0 |
| SNPs (1,2) | 2-1 | 7 | 2 | 5 | **SNPs (2,3)** | **2-1** | **8** | **17** | **-9** |
| SNPs (1,2) | 2-2 | 8 | 16 | -8 | SNPs (2,3) | 2-2 | 1 | 1 | 0 |
| SNPs (1,2) | 2-3 | 7 | 8 | -1 | SNPs (2,3) | 2-3 | 0 | 0 | 0 |
| SNPs (1,2) | 3-1 | 2 | 1 | 1 | SNPs (2,3) | 3-1 | 5 | 7 | -2 |
| SNPs (1,2) | 3-2 | 0 | 2 | -2 | SNPs (2,3) | 3-2 | 3 | 1 | 2 |
| SNPs (1,2) | 3-3 | 0 | 0 | 0 | SNPs (2,3) | 3-3 | 0 | 0 | 0 |
| SNPs (1,3) | 1-1 | 3 | 0 | 3 | SNPs (2,4) | 1-1 | 0 | 0 | 0 |
| SNPs (1,3) | 1-2 | 1 | 0 | 1 | SNPs (2,4) | 1-2 | 3 | 1 | 2 |
| SNPs (1,3) | 1-3 | 0 | 0 | 0 | SNPs (2,4) | 1-3 | 8 | 2 | 6 |
| SNPs (1,3) | 2-1 | 19 | 24 | -5 | SNPs (2,4) | 2-1 | 0 | 0 | 0 |
| SNPs (1,3) | 2-2 | 3 | 2 | 1 | SNPs (2,4) | 2-2 | 2 | 6 | -4 |
| SNPs (1,3) | 2-3 | 0 | 0 | 0 | SNPs (2,4) | 2-3 | 7 | 12 | -5 |
| SNPs (1,3) | 3-1 | 2 | 3 | -1 | SNPs (2,4) | 3-1 | 1 | 0 | 1 |
| SNPs (1,3) | 3-2 | 0 | 0 | 0 | SNPs (2,4) | 3-2 | 5 | 5 | 0 |
| SNPs (1,3) | 3-3 | 0 | 0 | 0 | SNPs (2,4) | 3-3 | 2 | 3 | -1 |
| SNPs (1,4) | 1-1 | 0 | 0 | 0 | SNPs (3,4) | 1-1 | 1 | 0 | 1 |
| SNPs (1,4) | 1-2 | 1 | 0 | 1 | SNPs (3,4) | 1-2 | 7 | 12 | -5 |
| SNPs (1,4) | 1-3 | 3 | 0 | 3 | SNPs (3,4) | 1-3 | 16 | 15 | 1 |
| SNPs (1,4) | 2-1 | 1 | 0 | 1 | SNPs (3,4) | 2-1 | 0 | 0 | 0 |
| SNPs (1,4) | 2-2 | 9 | 11 | -2 | SNPs (3,4) | 2-2 | 3 | 0 | 3 |
| SNPs (1,4) | 2-3 | 12 | 15 | -3 | SNPs (3,4) | 2-3 | 1 | 2 | -1 |
| SNPs (1,4) | 3-1 | 0 | 0 | 0 | SNPs (3,4) | 3-1 | 0 | 0 | 0 |
| SNPs (1,4) | 3-2 | 0 | 1 | -1 | SNPs (3,4) | 3-2 | 0 | 0 | 0 |
| SNPs (1,4) | 3-3 | 2 | 2 | 0 | SNPs (3,4) | 3-3 | 0 | 0 | 0 |

**Table S2. Results for two-SNP combinations**

Ca.: number of cases, Co.: number of controls, diff.: difference.

| **Combination of three SNPs** | **SNP**  **Genotype** | **Ca.** | **Co.** | **Diff.** | **Combination of three SNPs** | **SNP**  **Genotype** | **Ca.** | **Co.** | **Diff.** |
| --- | --- | --- | --- | --- | --- | --- | --- | --- | --- |
| SNPs(1,2,3) | 1-1-1 | 2 | 0 | 2 | SNPs(1,2,3) | 3-2-1 | 0 | 2 | -2 |
| SNPs(1,2,3) | 1-1-2 | 0 | 0 | 0 | SNPs(1,2,3) | 3-2-2 | 0 | 0 | 0 |
| SNPs(1,2,3) | 1-1-3 | 0 | 0 | 0 | SNPs(1,2,3) | 3-2-3 | 0 | 0 | 0 |
| SNPs(1,2,3) | 1-2-1 | 1 | 0 | 1 | SNPs(1,2,3) | 3-3-1 | 0 | 0 | 0 |
| SNPs(1,2,3) | 1-2-2 | 0 | 0 | 0 | SNPs(1,2,3) | 3-3-2 | 0 | 0 | 0 |
| SNPs(1,2,3) | 1-2-3 | 0 | 0 | 0 | SNPs(1,2,3) | 3-3-3 | 0 | 0 | 0 |
| SNPs(1,2,3) | 1-3-1 | 0 | 0 | 0 | SNPs(1,2,4) | 1-1-1 | 0 | 0 | 0 |
| SNPs(1,2,3) | 1-3-2 | 1 | 0 | 1 | SNPs(1,2,4) | 1-1-2 | 0 | 0 | 0 |
| SNPs(1,2,3) | 1-3-3 | 0 | 0 | 0 | SNPs(1,2,4) | 1-1-3 | 2 | 0 | 2 |
| **SNPs(1,2,3)** | **2-1-1** | **7** | **2** | **5** | SNPs(1,2,4) | 1-2-1 | 0 | 0 | 0 |
| SNPs(1,2,3) | 2-1-2 | 0 | 0 | 0 | SNPs(1,2,4) | 1-2-2 | 0 | 0 | 0 |
| SNPs(1,2,3) | 2-1-3 | 0 | 0 | 0 | SNPs(1,2,4) | 1-2-3 | 1 | 0 | 1 |
| **SNPs(1,2,3)** | **2-2-1** | **7** | **15** | **-8** | SNPs(1,2,4) | 1-3-1 | 0 | 0 | 0 |
| SNPs(1,2,3) | 2-2-2 | 1 | 1 | 0 | SNPs(1,2,4) | 1-3-2 | 1 | 0 | 1 |
| SNPs(1,2,3) | 2-2-3 | 0 | 0 | 0 | SNPs(1,2,4) | 1-3-3 | 0 | 0 | 0 |
| SNPs(1,2,3) | 2-3-1 | 5 | 7 | -2 | SNPs(1,2,4) | 2-1-1 | 0 | 0 | 0 |
| SNPs(1,2,3) | 2-3-2 | 2 | 1 | 1 | SNPs(1,2,4) | 2-1-2 | 3 | 1 | 2 |
| SNPs(1,2,3) | 2-3-3 | 0 | 0 | 0 | SNPs(1,2,4) | 2-1-3 | 4 | 1 | 3 |
| SNPs(1,2,3) | 3-1-1 | 2 | 1 | 1 | SNPs(1,2,4) | 2-2-1 | 0 | 0 | 0 |
| SNPs(1,2,3) | 3-1-2 | 0 | 0 | 0 | SNPs(1,2,4) | 2-2-2 | 2 | 5 | -3 |
| SNPs(1,2,3) | 3-1-3 | 0 | 0 | 0 | SNPs(1,2,4) | 2-2-3 | 6 | 11 | -5 |
| SNPs(1,2,3) | 1-1-1 | 2 | 0 | 2 | SNPs(1,2,4) | 2-3-1 | 1 | 0 | 1 |
| SNPs(1,2,3) | 1-1-2 | 0 | 0 | 0 | SNPs(1,2,4) | 3-1-1 | 0 | 0 | 0 |
| SNPs(1,2,3) | 1-1-3 | 0 | 0 | 0 | SNPs(1,2,4) | 3-1-2 | 0 | 0 | 0 |
| SNPs(1,2,3) | 1-2-1 | 1 | 0 | 1 | SNPs(1,2,4) | 3-1-3 | 2 | 1 | 1 |
| SNPs(1,2,3) | 1-2-2 | 0 | 0 | 0 | SNPs(1,2,4) | 3-2-1 | 0 | 0 | 0 |
| SNPs(1,2,3) | 1-2-3 | 0 | 0 | 0 | SNPs(1,2,4) | 3-2-2 | 0 | 1 | -1 |

**Table S3. Results for three-SNP combinations**

Ca.: number of cases, Co.: number of controls, Diff.: difference.

| **Combination of three SNPs** | **SNP**  **Genotype** | **Ca.** | **Co.** | **Diff.** | **Combination of three SNPs** | **SNP**  **Genotype** | **Ca.** | **Co.** | **Diff.** |
| --- | --- | --- | --- | --- | --- | --- | --- | --- | --- |
| SNPs(1,2,4) | 3-2-3 | 0 | 1 | -1 | SNPs(1,3,4) | 3-3-2 | 0 | 0 | 0 |
| SNPs(1,2,4) | 3-3-1 | 0 | 0 | 0 | SNPs(1,3,4) | 3-3-3 | 0 | 0 | 0 |
| SNPs(1,2,4) | 3-3-2 | 0 | 0 | 0 | SNPs(2,3,4) | 1-1-1 | 0 | 0 | 0 |
| SNPs(1,2,4) | 3-3-3 | 0 | 0 | 0 | SNPs(2,3,4) | 1-1-2 | 3 | 1 | 2 |
| SNPs(1,3,4) | 1-1-1 | 0 | 0 | 0 | SNPs(2,3,4) | 1-1-3 | 8 | 2 | 6 |
| SNPs(1,3,4) | 1-1-2 | 0 | 0 | 0 | SNPs(2,3,4) | 1-2-1 | 0 | 0 | 0 |
| SNPs(1,3,4) | 1-1-3 | 3 | 0 | 3 | SNPs(2,3,4) | 1-2-2 | 0 | 0 | 0 |
| SNPs(1,3,4) | 1-2-1 | 0 | 0 | 0 | SNPs(2,3,4) | 1-2-3 | 0 | 0 | 0 |
| SNPs(1,3,4) | 1-2-2 | 1 | 0 | 1 | SNPs(2,3,4) | 1-3-1 | 0 | 0 | 0 |
| SNPs(1,3,4) | 1-2-3 | 0 | 0 | 0 | SNPs(2,3,4) | 1-3-2 | 0 | 0 | 0 |
| SNPs(1,3,4) | 1-3-1 | 0 | 0 | 0 | SNPs(2,3,4) | 1-3-3 | 0 | 0 | 0 |
| SNPs(1,3,4) | 1-3-2 | 0 | 0 | 0 | SNPs(2,3,4) | 2-1-1 | 0 | 0 | 0 |
| SNPs(1,3,4) | 1-3-3 | 0 | 0 | 0 | SNPs(2,3,4) | 2-1-2 | 2 | 6 | -4 |
| SNPs(1,3,4) | 2-1-1 | 1 | 0 | 1 | SNPs(2,3,4) | 2-1-3 | 6 | 11 | -5 |
| SNPs(1,3,4) | 2-1-2 | 7 | 11 | -4 | SNPs(2,3,4) | 2-2-1 | 0 | 0 | 0 |
| SNPs(1,3,4) | 2-1-3 | 11 | 13 | -2 | SNPs(2,3,4) | 2-2-2 | 0 | 0 | 0 |
| SNPs(1,3,4) | 2-2-1 | 0 | 0 | 0 | SNPs(2,3,4) | 2-2-3 | 1 | 1 | 0 |
| SNPs(1,3,4) | 2-2-2 | 2 | 0 | 2 | SNPs(2,3,4) | 2-3-1 | 0 | 0 | 0 |
| SNPs(1,3,4) | 2-2-3 | 1 | 2 | -1 | SNPs(2,3,4) | 2-3-2 | 0 | 0 | 0 |
| SNPs(1,3,4) | 2-3-1 | 0 | 0 | 0 | SNPs(2,3,4) | 2-3-3 | 0 | 0 | 0 |
| SNPs(1,3,4) | 2-3-2 | 0 | 0 | 0 | SNPs(2,3,4) | 3-1-1 | 1 | 0 | 1 |
| SNPs(1,3,4) | 2-3-3 | 0 | 0 | 0 | SNPs(2,3,4) | 3-1-2 | 2 | 5 | -3 |
| SNPs(1,3,4) | 3-1-1 | 0 | 0 | 0 | SNPs(2,3,4) | 3-1-3 | 2 | 2 | 0 |
| SNPs(1,3,4) | 3-1-2 | 0 | 1 | -1 | SNPs(2,3,4) | 3-2-1 | 0 | 0 | 0 |
| SNPs(1,3,4) | 3-1-3 | 2 | 2 | 0 | SNPs(2,3,4) | 3-2-2 | 3 | 0 | 3 |
| SNPs(1,3,4) | 3-2-1 | 0 | 0 | 0 | SNPs(2,3,4) | 3-2-3 | 0 | 1 | -1 |
| SNPs(1,3,4) | 3-2-2 | 0 | 0 | 0 | SNPs(2,3,4) | 3-3-1 | 0 | 0 | 0 |
| SNPs(1,3,4) | 3-2-3 | 0 | 0 | 0 | SNPs(2,3,4) | 3-3-2 | 0 | 0 | 0 |
| SNPs(1,3,4) | 3-3-1 | 0 | 0 | 0 | SNPs(2,3,4) | 3-3-3 | 0 | 0 | 0 |

Ca.: number of cases, Co.: number of controls, Diff.: difference.

| **Combination of four SNPs** | **SNP**  **Genotype** | **Ca.** | **Co.** | **Diff.** | **Combination of four SNPs** | **SNP**  **Genotype** | **Ca.** | **Co.** | **Diff.** |
| --- | --- | --- | --- | --- | --- | --- | --- | --- | --- |
| SNPs(1,2,3,4) | 1-1-1-1 | 0 | 0 | 0 | SNPs(1,2,3,4) | 2-1-1-1 | 0 | 0 | 0 |
| SNPs(1,2,3,4) | 1-1-1-2 | 0 | 0 | 0 | SNPs(1,2,3,4) | 2-1-1-2 | 3 | 1 | 2 |
| SNPs(1,2,3,4) | 1-1-1-3 | 2 | 0 | 2 | **SNPs(1,2,3,4)** | **2-1-1-3** | **4** | **1** | **3** |
| SNPs(1,2,3,4) | 1-1-2-1 | 0 | 0 | 0 | SNPs(1,2,3,4) | 2-1-2-1 | 0 | 0 | 0 |
| SNPs(1,2,3,4) | 1-1-2-2 | 0 | 0 | 0 | SNPs(1,2,3,4) | 2-1-2-2 | 0 | 0 | 0 |
| SNPs(1,2,3,4) | 1-1-2-3 | 0 | 0 | 0 | SNPs(1,2,3,4) | 2-1-2-3 | 0 | 0 | 0 |
| SNPs(1,2,3,4) | 1-1-3-1 | 0 | 0 | 0 | SNPs(1,2,3,4) | 2-1-3-1 | 0 | 0 | 0 |
| SNPs(1,2,3,4) | 1-1-3-2 | 0 | 0 | 0 | SNPs(1,2,3,4) | 2-1-3-2 | 0 | 0 | 0 |
| SNPs(1,2,3,4) | 1-1-3-3 | 0 | 0 | 0 | SNPs(1,2,3,4) | 2-1-3-3 | 0 | 0 | 0 |
| SNPs(1,2,3,4) | 1-2-1-1 | 0 | 0 | 0 | SNPs(1,2,3,4) | 2-2-1-1 | 0 | 0 | 0 |
| SNPs(1,2,3,4) | 1-2-1-2 | 0 | 0 | 0 | SNPs(1,2,3,4) | 2-2-1-2 | 2 | 5 | -3 |
| SNPs(1,2,3,4) | 1-2-1-3 | 1 | 0 | 1 | **SNPs(1,2,3,4)** | **2-2-1-3** | **5** | **10** | **-5** |
| SNPs(1,2,3,4) | 1-2-2-1 | 0 | 0 | 0 | SNPs(1,2,3,4) | 2-2-2-1 | 0 | 0 | 0 |
| SNPs(1,2,3,4) | 1-2-2-2 | 0 | 0 | 0 | SNPs(1,2,3,4) | 2-2-2-2 | 0 | 0 | 0 |
| SNPs(1,2,3,4) | 1-2-2-3 | 0 | 0 | 0 | SNPs(1,2,3,4) | 2-2-2-3 | 1 | 1 | 0 |
| SNPs(1,2,3,4) | 1-2-3-1 | 0 | 0 | 0 | SNPs(1,2,3,4) | 2-2-3-1 | 0 | 0 | 0 |
| SNPs(1,2,3,4) | 1-2-3-2 | 0 | 0 | 0 | SNPs(1,2,3,4) | 2-2-3-2 | 0 | 0 | 0 |
| SNPs(1,2,3,4) | 1-2-3-3 | 0 | 0 | 0 | SNPs(1,2,3,4) | 2-2-3-3 | 0 | 0 | 0 |
| SNPs(1,2,3,4) | 1-3-1-1 | 0 | 0 | 0 | SNPs(1,2,3,4) | 2-3-1-1 | 1 | 0 | 1 |
| SNPs(1,2,3,4) | 1-3-1-2 | 0 | 0 | 0 | SNPs(1,2,3,4) | 2-3-1-2 | 2 | 5 | -3 |
| SNPs(1,2,3,4) | 1-3-1-3 | 0 | 0 | 0 | SNPs(1,2,3,4) | 2-3-1-3 | 2 | 2 | 0 |
| SNPs(1,2,3,4) | 1-3-2-1 | 0 | 0 | 0 | SNPs(1,2,3,4) | 2-3-2-1 | 0 | 0 | 0 |
| SNPs(1,2,3,4) | 1-3-2-2 | 1 | 0 | 1 | SNPs(1,2,3,4) | 2-3-2-2 | 2 | 0 | 2 |
| SNPs(1,2,3,4) | 1-3-2-3 | 0 | 0 | 0 | SNPs(1,2,3,4) | 2-3-2-3 | 0 | 1 | -1 |
| SNPs(1,2,3,4) | 1-3-3-1 | 0 | 0 | 0 | SNPs(1,2,3,4) | 2-3-3-1 | 0 | 0 | 0 |
| SNPs(1,2,3,4) | 1-3-3-2 | 0 | 0 | 0 | SNPs(1,2,3,4) | 2-3-3-2 | 0 | 0 | 0 |
| SNPs(1,2,3,4) | 1-3-3-3 | 0 | 0 | 0 | SNPs(1,2,3,4) | 2-3-3-3 | 0 | 0 | 0 |

**Table S4. Results for four-SNP combinations**

Ca.: number of cases, Co.: number of controls, Diff.: difference.

| **Combination of four SNPs** | **SNP**  **Genotype** | **Ca.** | **Co.** | **Diff.** | **Combination of four SNPs** | **SNP**  **Genotype** | **Ca.** | **Co.** | **Diff.** |
| --- | --- | --- | --- | --- | --- | --- | --- | --- | --- |
| SNPs(1,2,3,4) | 3-1-1-1 | 0 | 0 | 0 | SNPs(1,2,3,4) | 3-2-2-1 | 0 | 0 | 0 |
| SNPs(1,2,3,4) | 3-1-1-2 | 0 | 0 | 0 | SNPs(1,2,3,4) | 3-2-2-2 | 0 | 0 | 0 |
| SNPs(1,2,3,4) | 3-1-1-3 | 2 | 1 | 1 | SNPs(1,2,3,4) | 3-2-2-3 | 0 | 0 | 0 |
| SNPs(1,2,3,4) | 3-1-2-1 | 0 | 0 | 0 | SNPs(1,2,3,4) | 3-3-1-1 | 0 | 0 | 0 |
| SNPs(1,2,3,4) | 3-1-2-2 | 0 | 0 | 0 | SNPs(1,2,3,4) | 3-3-1-2 | 0 | 0 | 0 |
| SNPs(1,2,3,4) | 3-1-2-3 | 0 | 0 | 0 | SNPs(1,2,3,4) | 3-3-1-3 | 0 | 0 | 0 |
| SNPs(1,2,3,4) | 3-1-3-1 | 0 | 0 | 0 | SNPs(1,2,3,4) | 3-3-2-1 | 0 | 0 | 0 |
| SNPs(1,2,3,4) | 3-1-3-2 | 0 | 0 | 0 | SNPs(1,2,3,4) | 3-3-2-2 | 0 | 0 | 0 |
| SNPs(1,2,3,4) | 3-1-3-3 | 0 | 0 | 0 | SNPs(1,2,3,4) | 3-3-2-3 | 0 | 0 | 0 |
| SNPs(1,2,3,4) | 3-2-1-1 | 0 | 0 | 0 | SNPs(1,2,3,4) | 3-3-3-1 | 0 | 0 | 0 |
| SNPs(1,2,3,4) | 3-2-1-2 | 0 | 1 | -1 | SNPs(1,2,3,4) | 3-3-3-2 | 0 | 0 | 0 |
| SNPs(1,2,3,4) | 3-2-1-3 | 0 | 1 | -1 | SNPs(1,2,3,4) | 3-3-3-3 | 0 | 0 | 0 |

Ca.: number of cases, Co.: number of controls, Diff.: difference.

**Table S5. Table of cases and controls**

|  | Case | Control |
| --- | --- | --- |
| Risk Factor Present  (Positive) | TP - 8 | FP -17 |
| Risk Factor Absent  (Negative) | FN - 20 | TN -13 |

| $CC=\frac{TP+TN}{TP+FN+FP+TN}$ | (1) |
| --- | --- |
| $SN=\frac{TP}{TP+FN}$ | (2) |
| $SP=\frac{TN}{TN+FP}$ | (3) |
| $OR=\frac{TP\times TN}{FN\times FP}$ | (4) |

**Table S6. Common criteria**

**Table S7. Performance calculation**

|  |
| --- |
|  |
|  |
|  |
